# Supplementary material for: Novel Driver Strength Index highlights important cancer genes in TCGA PanCanAtlas patients
Source: PeerJ. 2022 Aug 11;10:e13860. doi: 10.7717/peerj.13860 (PMC9375969; doi:10.7717/peerj.13860)
Supplement: Supplemental Information 2 [file peerj-10-13860-s002.zip › COHORTS/genes plots/detailed/2021_8_16_14_9_distribution_events_detailed_males_43.pdf]

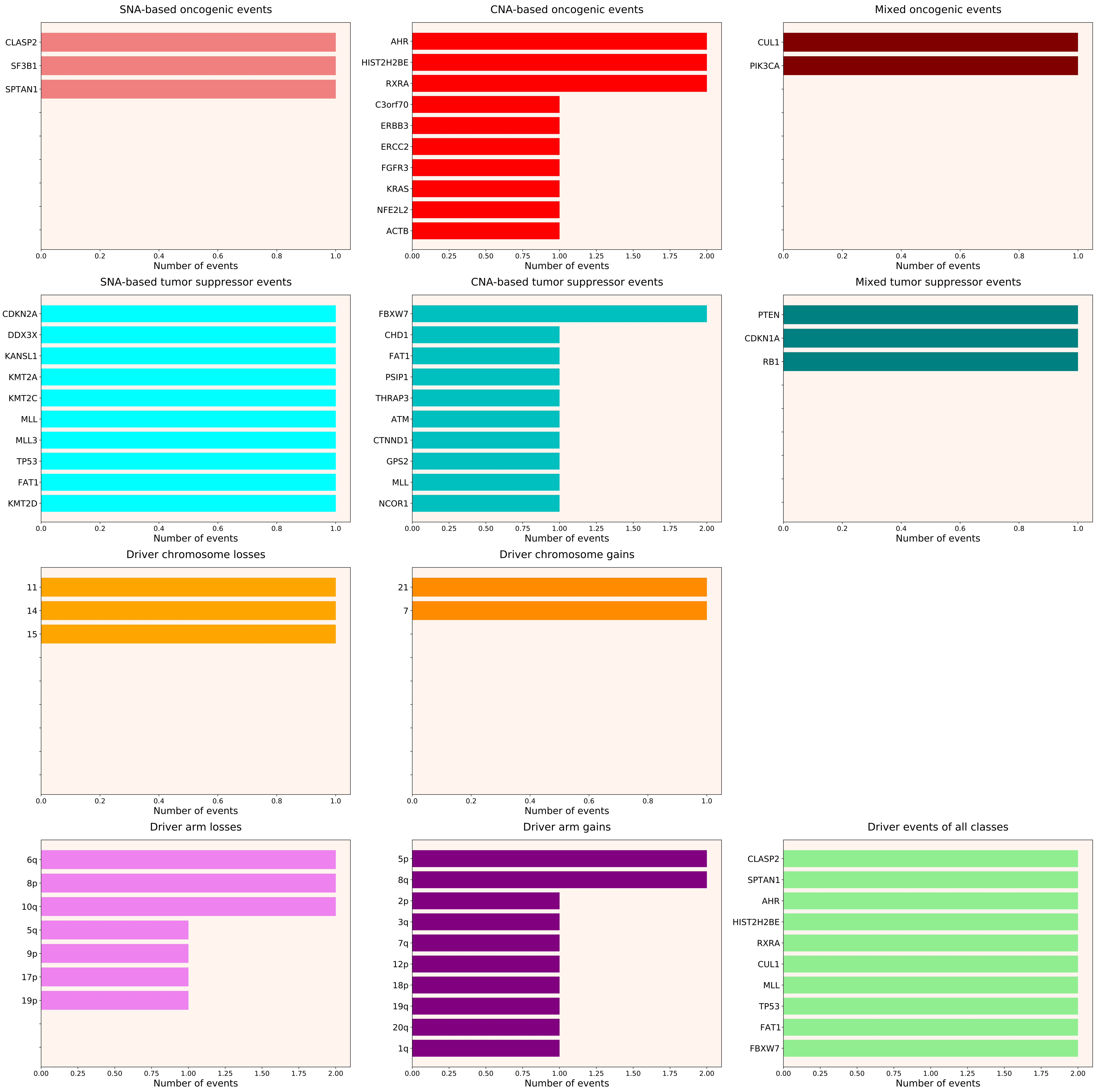
SNA-based oncogenic events
CLASP2
SF3B1
SPTAN1
Number of events
CNA-based oncogenic events
AHR
HIST2H2BE
RXRA
C3orf70
ERBB3
ERCC2
FGFR3
KRAS
NFE2L2
ACTB
Number of events
Mixed oncogenic events
CUL1
PIK3CA
Number of events
SNA-based tumor suppressor events
CDKN2A
DDX3X
KANSL1
KMT2A
KMT2C
MLL
MLL3
TP53
FAT1
KMT2D
Number of events
CNA-based tumor suppressor events
FBXW7
CHD1
FAT1
PSIP1
THRAP3
ATM
CTNND1
GPS2
MLL
NCOR1
Number of events
Mixed tumor suppressor events
PTEN
CDKN1A
RB1
Number of events
Driver chromosome losses
11
14
15
Number of events
Driver chromosome gains
21
7
Number of events
Driver arm losses
6q
8p
10q
5q
9p
17p
19p
Number of events
Driver arm gains
5p
8q
2p
3q
7q
12p
18p
19q
20q
1q
Number of events
Driver events of all classes
CLASP2
SPTAN1
AHR
HIST2H2BE
RXRA
CUL1
MLL
TP53
FAT1
FBXW7
Number of events
